# Molecular and physiological mechanisms of tea (*Camellia sinensis* (L.) O. Kuntze) leaf and root in response to nitrogen deficiency

**DOI:** 10.1186/s12864-023-09112-y

**Published:** 2023-01-17

**Authors:** Zheng-He Lin, Chang-Song Chen, Shui-Qing Zhao, Yuan Liu, Qiu-Sheng Zhong, Qi-Chun Ruan, Zhi-Hui Chen, Xiao-Mei You, Rui-Yang Shan, Xin-Lei Li, Ya-Zhen Zhang

**Affiliations:** 1 Tea Research Institute, Fujian Academy of Agricultural Sciences, Fu’an, 355000 China; 2 Laixi Bureau of Agriculture and Rural Affairs of Shandong Province, Laixi, 266699 China

**Keywords:** Tea, Nitrogen deficiency, Nitrogen metabolism, Transcriptome profile

## Abstract

**Background:**

As an economically important crop, tea is strongly nitrogen (N)-dependent. However, the physiological and molecular mechanisms underlying the response of N deficiency in tea are not fully understood. Tea cultivar “Chunlv2” [*Camellia sinensis* (L.) O. Kuntze] were cultured with a nutrient solution with 0 mM [N-deficiency] or 3 mM (Control) NH_4_NO_3_ in 6 L pottery pots containing clean river sands.

**Results:**

N deficiency significantly decreased N content, dry weight, chlorophyll (Chl) content, L-theanine and the activities of N metabolism-related enzymes, but increased the content of total flavonoids and polyphenols in tea leaves. N deficiency delayed the sprouting time of tea buds. By using the RNA-seq technique and subsequent bioinformatics analysis, 3050 up-regulated and 2688 down-regulated differentially expressed genes (DEGs) were isolated in tea leaves in response to N deficiency. However, only 1025 genes were up-regulated and 744 down-regulated in roots. Gene ontology (GO) term enrichment analysis showed that 205 DEGs in tea leaves were enriched in seven GO terms and 152 DEGs in tea roots were enriched in 11 GO items based on *P* < 0.05. In tea leaves, most GO-enriched DEGs were involved in chlorophyll a/b binding activities, photosynthetic performance, and transport activities. But most of the DEGs in tea roots were involved in the metabolism of carbohydrates and plant hormones with regard to the GO terms of biological processes. N deficiency significantly increased the expression level of phosphate transporter genes, which indicated that N deficiency might impair phosphorus metabolism in tea leaves. Furthermore, some DEGs, such as probable anion transporter 3 and high-affinity nitrate transporter 2.7, might be of great potential in improving the tolerance of N deficiency in tea plants and further study could work on this area in the future.

**Conclusions:**

Our results indicated N deficiency inhibited the growth of tea plant, which might be due to altered N metabolism and expression levels of DEGs involved in the photosynthetic performance, transport activity and oxidation–reduction processes.

**Supplementary Information:**

The online version contains supplementary material available at 10.1186/s12864-023-09112-y.

## Background

Nitrogen (N) is one of the main factors limiting plant growth, and is also an essential component of nucleic acids, amino acids, proteins, chlorophyll and some plant hormones. Tea is an important economic crop, and is rich in many secondary metabolites that are closely related with its quality and health functions [1]. The growth and development of tea plant demand more nitrogen than other crops, and tea leaf quality was greatly affected by the nitrogen component [2, 3]. However, the molecular mechanism underlying N-use efficiency of tea plants was remained obscure. Along with the fresh leaves being picked, a large amount of nitrogen was taken away, in order to gain high quality, high yield and incomes in tea industry, nitrogen fertilizer was heavily applied in tea gardens of Southern China [4]. Existing studies indicated that the average amount of pure nitrogen applied to tea gardens in China was as high as 737.7 kg·hm^−2^ [4, 5]. It was estimated that more than half of N was lost from the plant soil system, and resulted in severe pollution of the environment [6]. Therefore, it is necessary to increase the N-use efficiency in order to cut pollution and increase productivity.

N-deficiency will lead to tea plant growth retardation and significantly decreased yield and quality of tea products [4]. Under N-deficiency, the thickness of leaves become thin and the leaves turn yellow, leading to the corresponding decrease of dry matter accumulation in tea plants. Available literatures indicated, altered expression levels of various genes associated with N-deficiency sustained plant growth and development by altering the level of chlorophyll synthesis [7], root architecture [8], improving N-assimilation [9], enhancing lignin content [10], and changing the amounts of soluble carbohydrates and sugar phosphates [11]. One of the conspicuous symptoms of N-deficiency was the chlorosis of plant leaves, which was due to the degradation of chlorophyll. Furthermore, N is a critical constituent in RuBisCO protein, the Calvin–Benson cycle related enzymes, and carotenoids in plant leaves [12]. N-deficiency-induced reduction of photosynthesis has been found in many crops including sunflower [13], rice [14], olive [15], and corn [16].

By using RNA-seq technique, Lian et al. [17] found that a total of 473 responsive genes were identified in the root system, among which, 115 up-regulated and 358 down-regulated under low nitrogen stress. Yang et al.[6] reported that 1,650 transcripts were differentially expressed (fold-change ≥ 2) in rice under N-deficiency. Among them, 1,158 were differentially expressed in the leaf sheaths (548 up-regulated and 610 down-regulated) and 492 in the roots (276 up, 216 down). There were 342 differentially expressed genes in nitrogen-deficient maize, most of which were related to amino acid metabolism, photosynthesis, secondary metabolism, gene replication and expression [18].

Generally speaking, plant nitrogen metabolism mainly includes nitrogen uptake, reduction, amino acid metabolism and transport, translocation and remobilization of nitrogen[19]. To be incorporated into amino acids and peptides, nitrate is reduced, converted and incorporated into glutamate in succession, by a cascade of enzymes including nitrate reductase (NR) in the cell cytosol, nitrite reductase (NiR) in plant plastids or leaf chloroplasts, and Glutamine synthetase-glutamate synthetase (GS-GOGAT) cycle in plants [20]. Many environment factors, such as nitrate supply, amino acid level, and sugar level, were reported to regulate NR and NiR activities [21, 22]. Previous study showed that the enzyme activities of NR, GS and GOGAT were decreased by decreasing N supply in citrus [23]. Over-expression of GS1 in tobacco and maize resulted in a significant increase in the plant heights, dry weights or kernel numbers [24, 25]. Over-expression of NADH-dependent GOGAT in rice and alanine amino-transferase in canola and rice also induce an increase of grain weights and biomass [26].Therefore, exploring the molecular and physiological basis of responses to nitrogen starvation in *Camellia sinensis* (L.) will contribute to the understanding of the mechanisms of N metabolism in plants.

In this study, we conducted a pot experiment to investigate the effects of N-deficiency on the growth, nitrogen metabolism and related genes expression in leaves of tea plants. The results of this experiment will be not only to provide a better understanding of the growth and development in tea plant response to N-deficiency, but also to offer a guiding technology of nitrogen management in tea garden, and promote an economic and sustainable agriculture for tea industry.

## Results

### Effects of N-deficiency on spring shoot growth and dry weight of tea plants

**Fig. 1:**
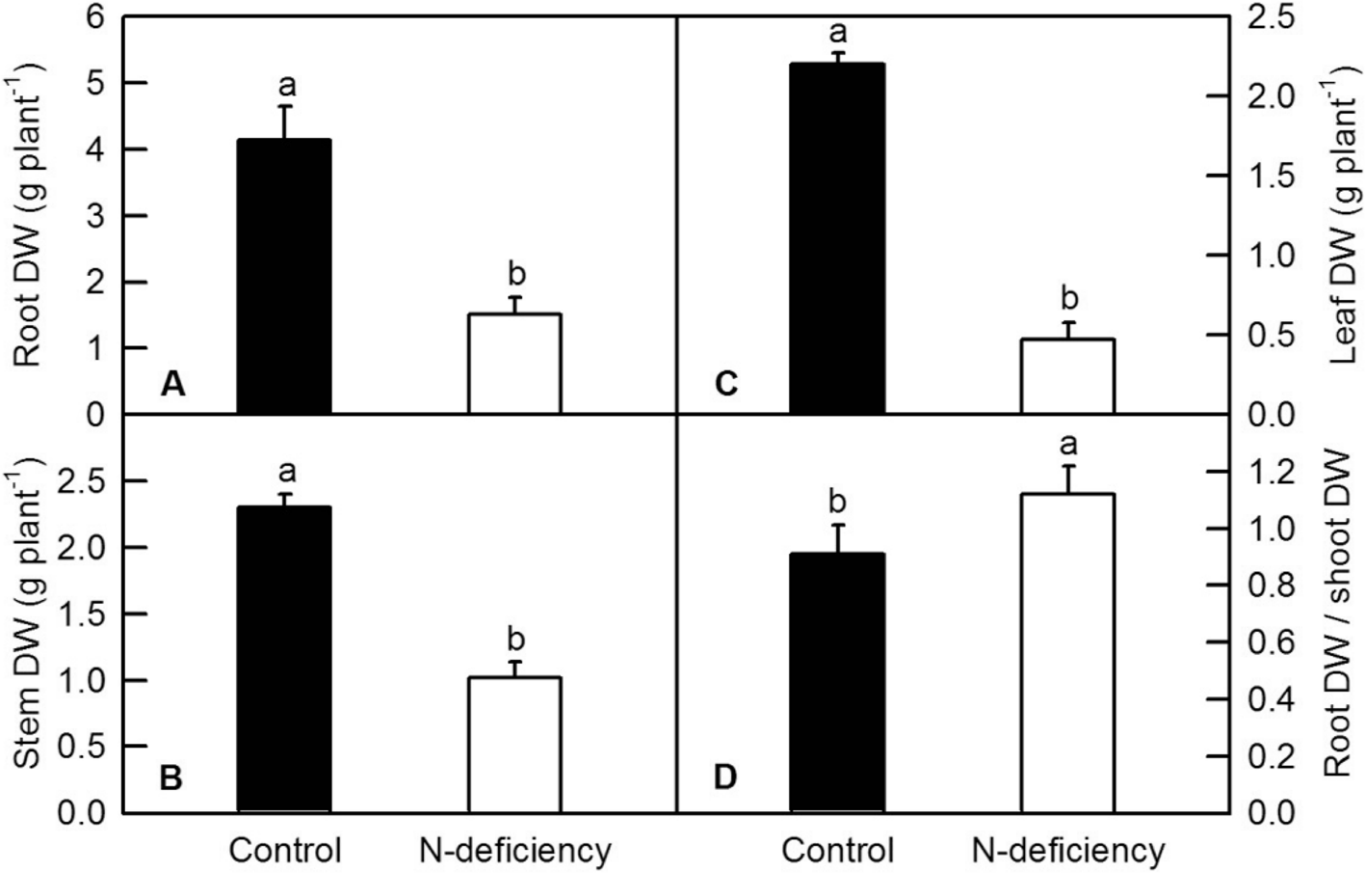
Effects of N-deficiency on root DW (**A**), stem DW (**B**), leaf DW (**C**) and ratio of root DW to shoot DW (root DW/shoot DW, **D**) of tea plants. The means of data were separated by student’s *t*-test, and bars were represented by mean ± standard error (SE, *n* = 6). Different letters above the bars indicated significant difference at* P* < 0.05

N-deficiency significantly delayed the sprouting of tea shoots. One bud with one leaf, one bud with the second leaf and one bud with the third leaf were delayed by 4, 11 and 13 days, respectively (Table 1). Also, N-deficiency significantly decreased the dry weight of root (Fig. 1A), stem (Fig. 1B) and leaf (Fig. 1C). N-deficiency significantly increased the ratio of root DW/shoot DW in tea plants (Fig. 1D).

**Table 1:** Effect of N-deficiency on the shoot growth of tea plants

Treatments	Date of one bud with the first leaf	Date of one bud with second leaf	Date of one bud with the third leaf
Control	8-Mar	12-Mar	14-Mar
N-deficiency	12-Mar	23-Mar	27-Mar
Intervals between with the treatments	4 days	11 days	13 days

### Effects of N-deficiency on total nitrogen content and activities of related enzymes tea plants

**Fig. 2:**
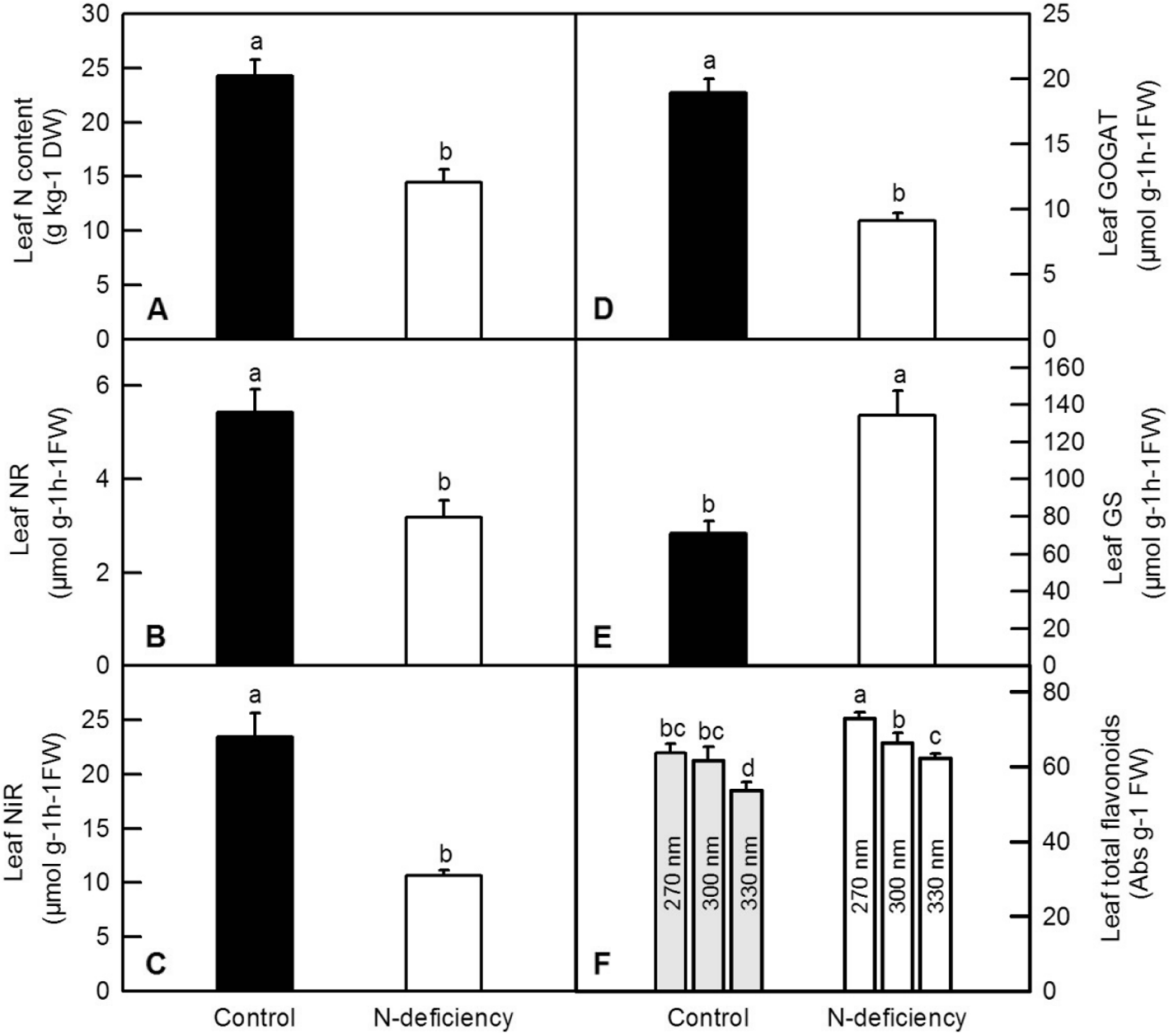
The effect of N-deficiency on leaf N content (**A**), leaf NR activity (**B**),leaf NiR activity (**C**), leaf GOGAT activity (**D**), leaf GS activity (**E**) and total flavonoids content (**F**) of tea plants. The means of data were separated by student’s *t*-test or least significant difference(LSD) test, and bars were represented by mean ± standard error (SE, *n* = 6). Different letters above the bars indicated significant difference at* P* < 0.05

**Fig. 3:**
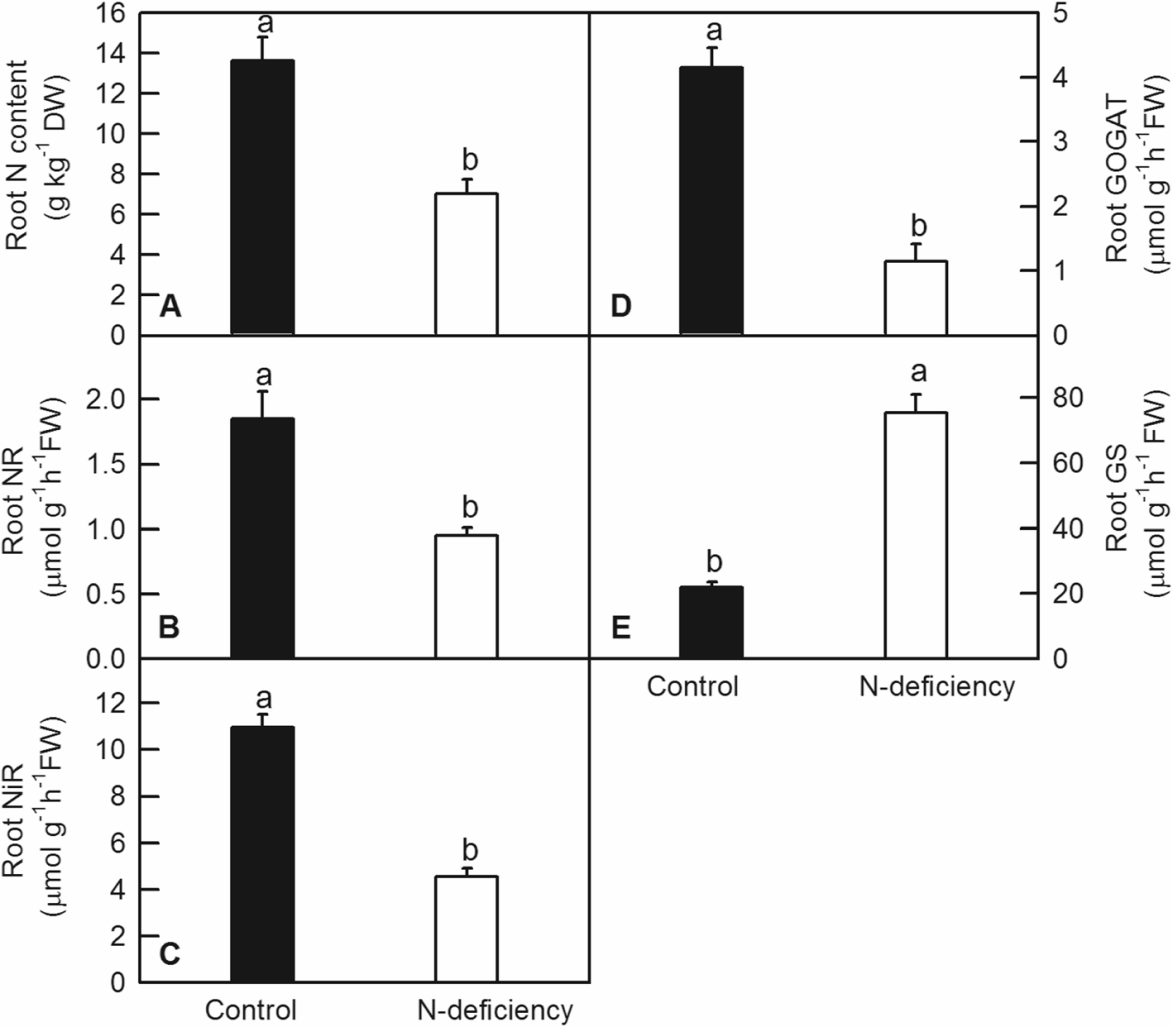
The effect of N-deficiency on root N content (**A**), root NR activity (**B**), root NiR activity (**C**), root GOGAT activity (**D**), and root GS activity (**E**) of tea plants. The means of data were separated by student’s *t*-test, and bars were represented by mean ± standard error (SE, *n* = 6). Different letters above the bars indicated significant difference at *P* < 0.05

NR, NiR, GOGAT and GS were involved in the uptake, ammonification and assimilation in amino acids in plant. Enzyme dynamic measurement found that N-deficiency significantly reduced leaf and root N content (Figs. 2A and 3A), the activities of GOGAT (Figs. 2D and 3D), NR (Figs. 2B and 3B) and NiR (Figs. 2C and 3C) in tea leaf and root, whereas remarkably elevated the activities of leaf GS and root GS (Figs. 2E and 3E).

### Effects of N-deficiency on the contents of chlorophyll and total flavonoids in tea leaves

N-deficiency significantly increased total flavonoids content represented as the UV-absorbing compounds at 270 nm and 330 nm wavelength in tea leaves, but did not change that value at 300 nm wavelength (Fig. 2F).

**Fig. 4:**
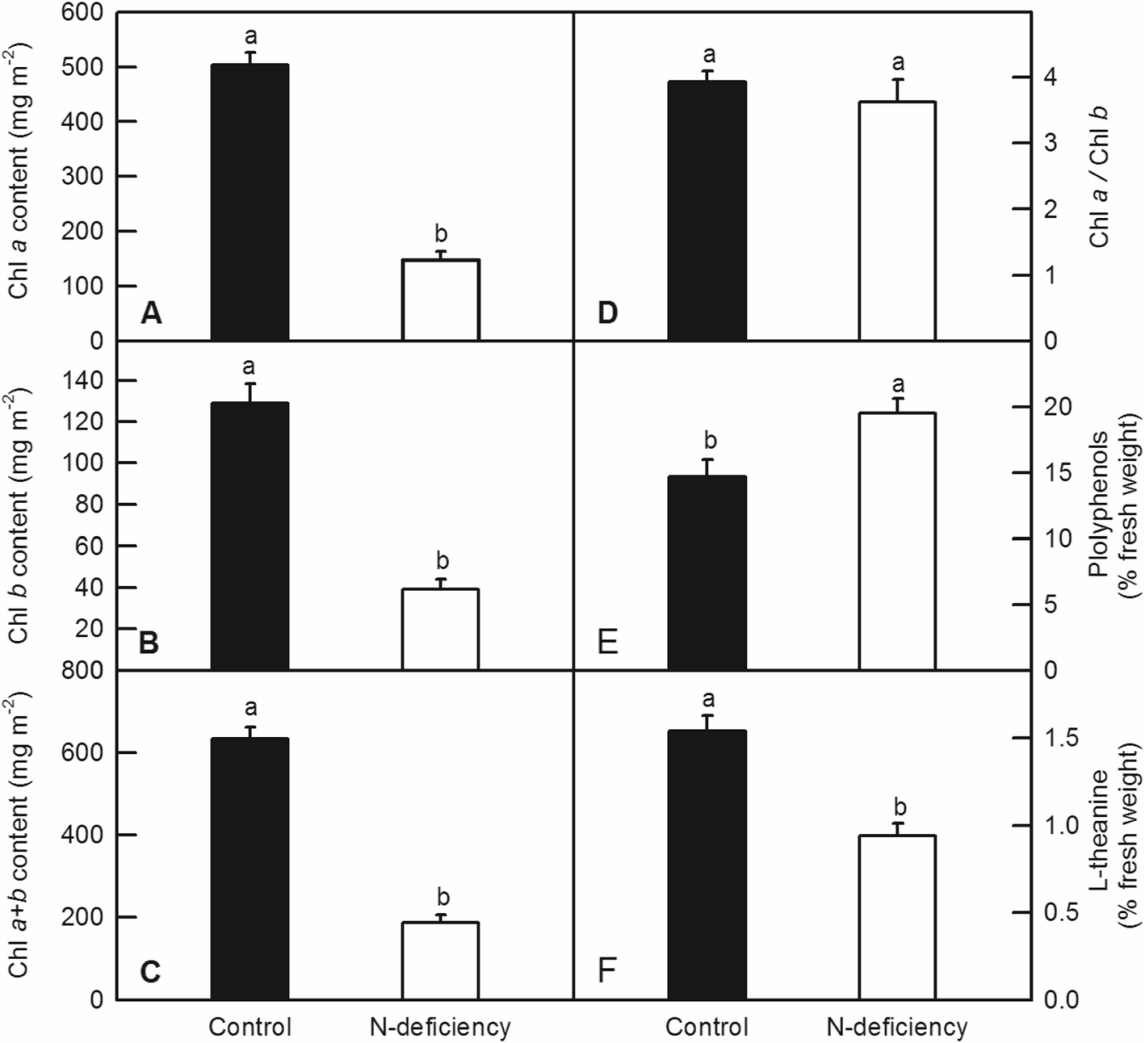
Effects of N-deficiency on the contents of Chl *a* (**A**), Chl *b* (**B**), Chl *a* + *b* (**C**), polyphenols (**E**), L-theanine (**F**) and the ratio of Chl *a*/Chl *b* (**D**) in tea leaves. The means of data were separated by student’s *t*-test, and bars were represented by mean ± standard error (SE, *n* = 6). Different letters above the bars indicated significant difference at *P* < 0.05

N-deficiency remarkedly decreased Chl *a* (Fig. 4A), Chl *b* (Fig. 4B) and total Chl (Chl *a* + *b*) contents (Fig. 4C). There was no significant change observed in the ratio of Chl*a* to Chl*b* (Chl *a*/*b*) (Fig. 4D).N-deficiency significantly increased the contents of polyphenols, whereas decreased the content of L-theanine in tea leaves (Fig. 4E and F).

### RNA-seq, de novo assembly, transcripts annotation and differentially expressed genes (DEGs) identification

Transcript profiles of the RNA-Seq data were analyzed by calculating the reads per kilo base per million reads (RPKM). Twelve cDNA libraries, including three biological replicates for normal nitrogen (CK) leaf (CKL1, CKL2 and CKL3), N-deficiency leaf (NNL1, NNL2 and NNL3), normal nitrogen (CK) root (CKR1, CKR2 and CKR3), N-deficiency root (NNR1, NNR2 and NNR3) were constructed and sequenced. The numbers of raw reads generated from each library ranged from 38,701,550 to 53,027,440. The percentages of clean reads and Q20 (sequencing error rates lower than 1%) were more than 98 and 95%, respectively (Table 2). Here, 77.57%-86.40% of the clean reads were mapped uniquely to the tea genome, and only a small proportion of them were mapped multiply to the genome [1]. The number of known transcripts generated from reference genomes ranged from 26,930 to 29,188, which accounted for 64.37–69.77% of the number of annotated genes in the genome. The RNA-Seq data was deposited in NCBI database (https://www.ncbi.nlm.nih.gov/sra/) with SRA accession number PRJNA747801.

**Table 2:** Summary of the RNA-Seq data of the control and N-deficient tea leaves and roots

Sample and treatment	Raw reads	Clean reads (%)	GC %	Q20	Q30
CKL1	44,099,018	43,547,360(98.75%)	44.05%	96.28%	90.97%
CKL2	43,366,334	42,740,292(98.56%)	44.16%	96.30%	91.09%
CKL3	46,852,726	46,505,542(99.26%)	43.92%	96.08%	90.59%
Average (CKL)	44,772,693	44,264,398(98.86%)	44.04%	96.22%	90.88%
NNL1	48,563,670	48,284,164(99.42%)	43.18%	95.78%	90.06%
NNL2	45,788,340	45,540,402(99.46%)	43.17%	95.99%	90.44%
NNL3	38,701,550	38,451,384(99.35%)	43.23%	96.19%	90.81%
Average (NNL)	44,351,187	44,091,983(99.42%)	43.19%	95.99%	90.44%
CKR1	44,600,070	44,324,964(99.38%)	43.72%	95.88%	90.28%
CKR2	42,203,314	41,834,642(99.13%)	43.72%	95.77%	90.02%
CKR3	53,027,440	52,667,716(99.32%)	42.97%	96.19%	91.01%
Average (CKR)	46,610,275	46,275,774(99.28%)	43.47%	95.95%	90.44%
NNR1	45,849,948	45,485,956(99.30%)	43.26%	96.13%	90.73%
NNR2	43,861,456	43,555,094(99.30%)	43.08%	95.87%	90.24%
NNR3	42,040,232	41,564,386(98.87%)	43.32%	95.86%	90.21%
Average (NNR)	43,917,212	43,535,145(99.13%)	43.22%	95.95%	90.39%

**Fig. 5:**
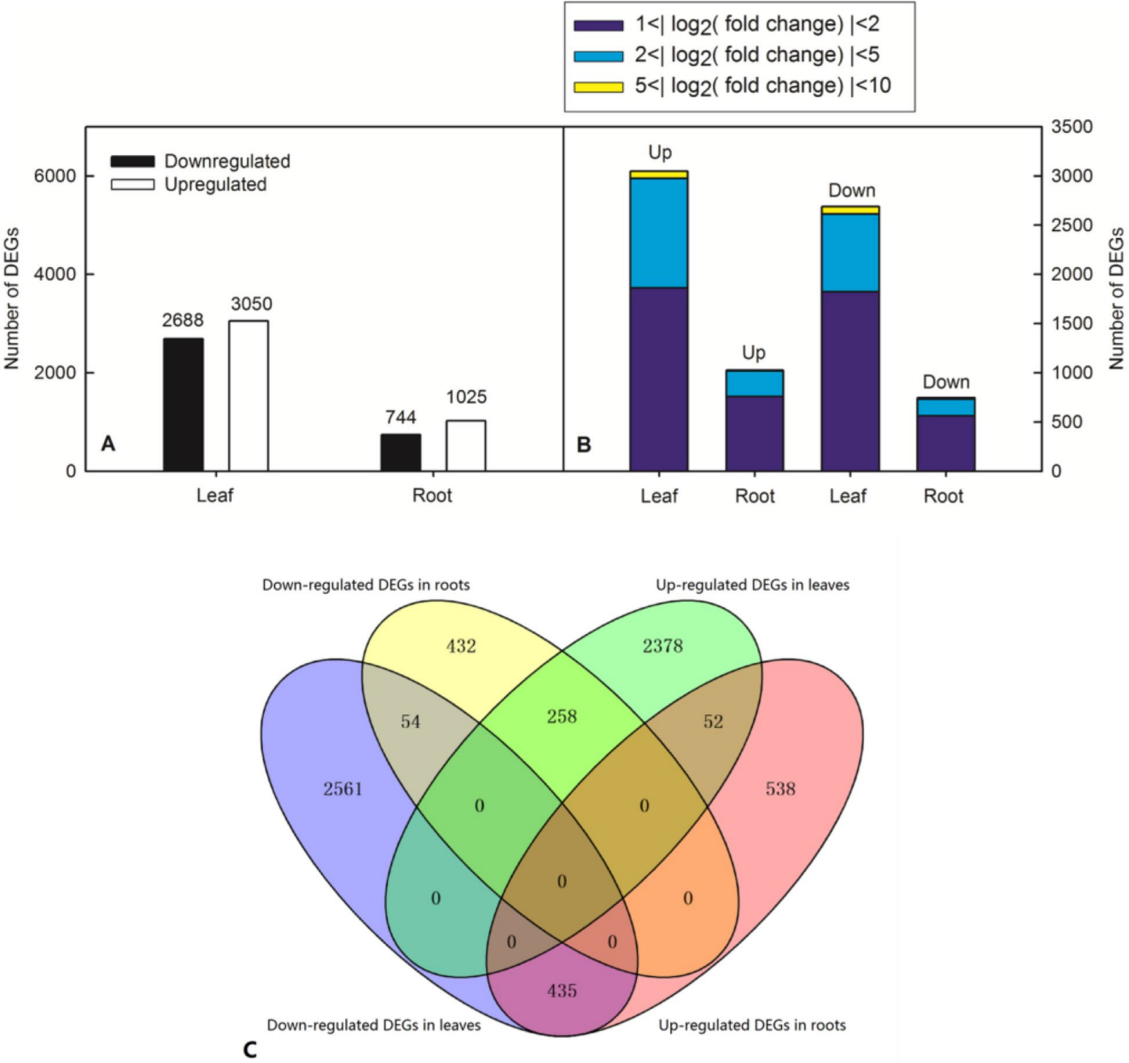
Effects of N-deficiency on DEGs identified in tea plant. **A** up-regulated and down-regulated genes in tea leaf and root under N-deficiency, **B** columnar diagram of N-responsive genes in tea leaf and root under N-deficiency

In this study, the DEGs between control and N-deficiency were identified with an absolute value of the log2 ratio (|log2(fold change)|) ≥ 1 and a threshold of FDR ≤ 0.05. We obtained 3050 up-regulated and 2688 down-regulated DEGs in tea leaf and 1025 up-regulated and 744 down-regulated DEGs in tea root (Fig. 5A; Table S1-S4 (Supplementary Materials)). Among these DEGs, the largest number of which was the DEGs with the |log_2_(fold change)| between 1 to 2, followed by 2 to 5 and 5 to 10, respectively (Fig. 5B). Venn diagram showed that there had 54 common DEGs between the down-regulated DEGs in tea leaves and tea roots,52 common DEGs between the up-regulated DEGs in tea leaves and tea roots, 258 common DEGs between the down-regulated DEGs in tea roots and up-regulated DEGs in tea leaves, and 435 common DEGs between the down-regulated DEGs in tea leaves and up-regulated DEGs in tea roots, respectively (Fig. 5C).

**Fig. 6:**
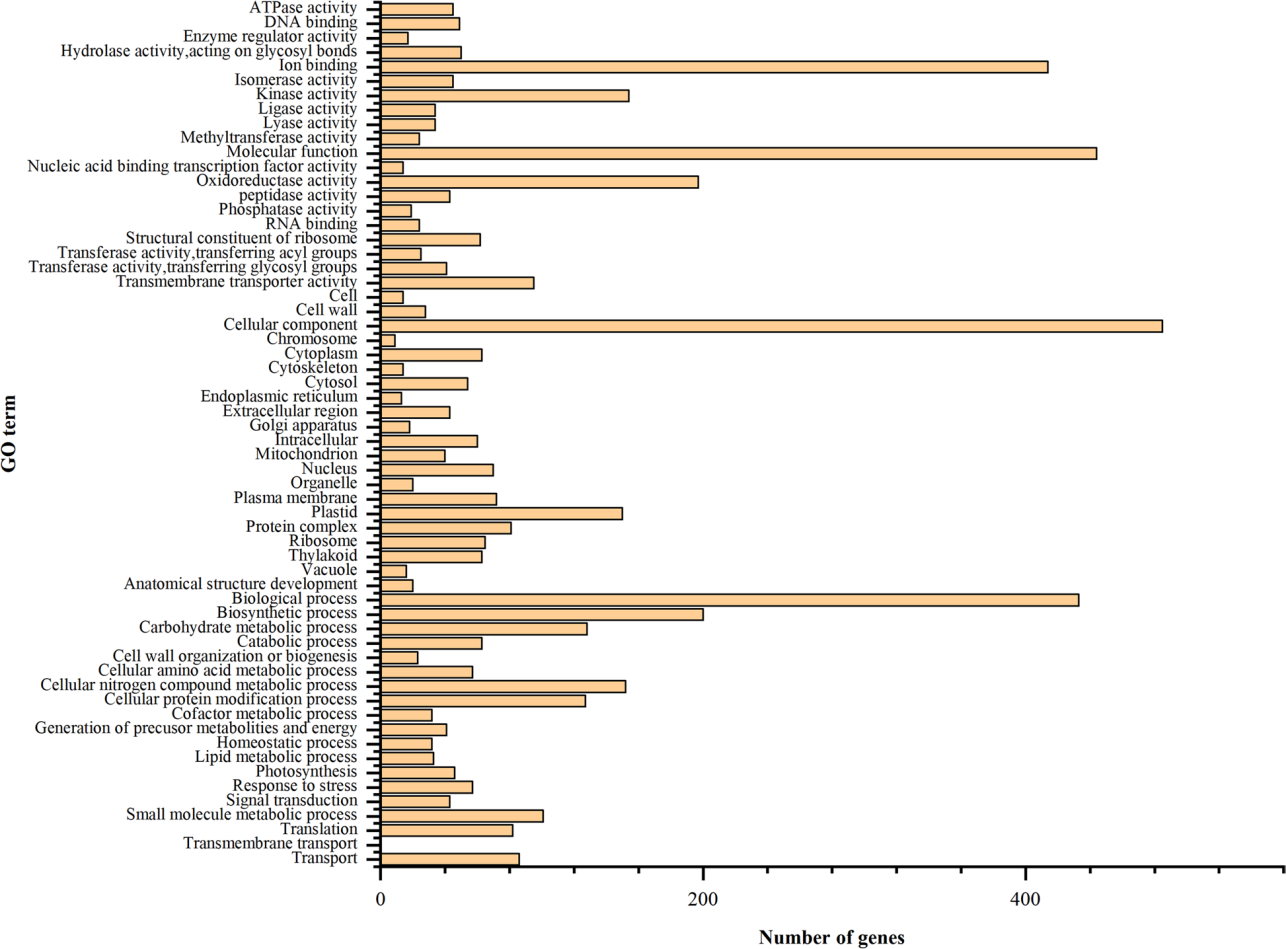
The most significantly-enriched GO terms of the differentially expressed genes from the four comparison groups: **A**: NNL vs CKL

**Fig. 7:**
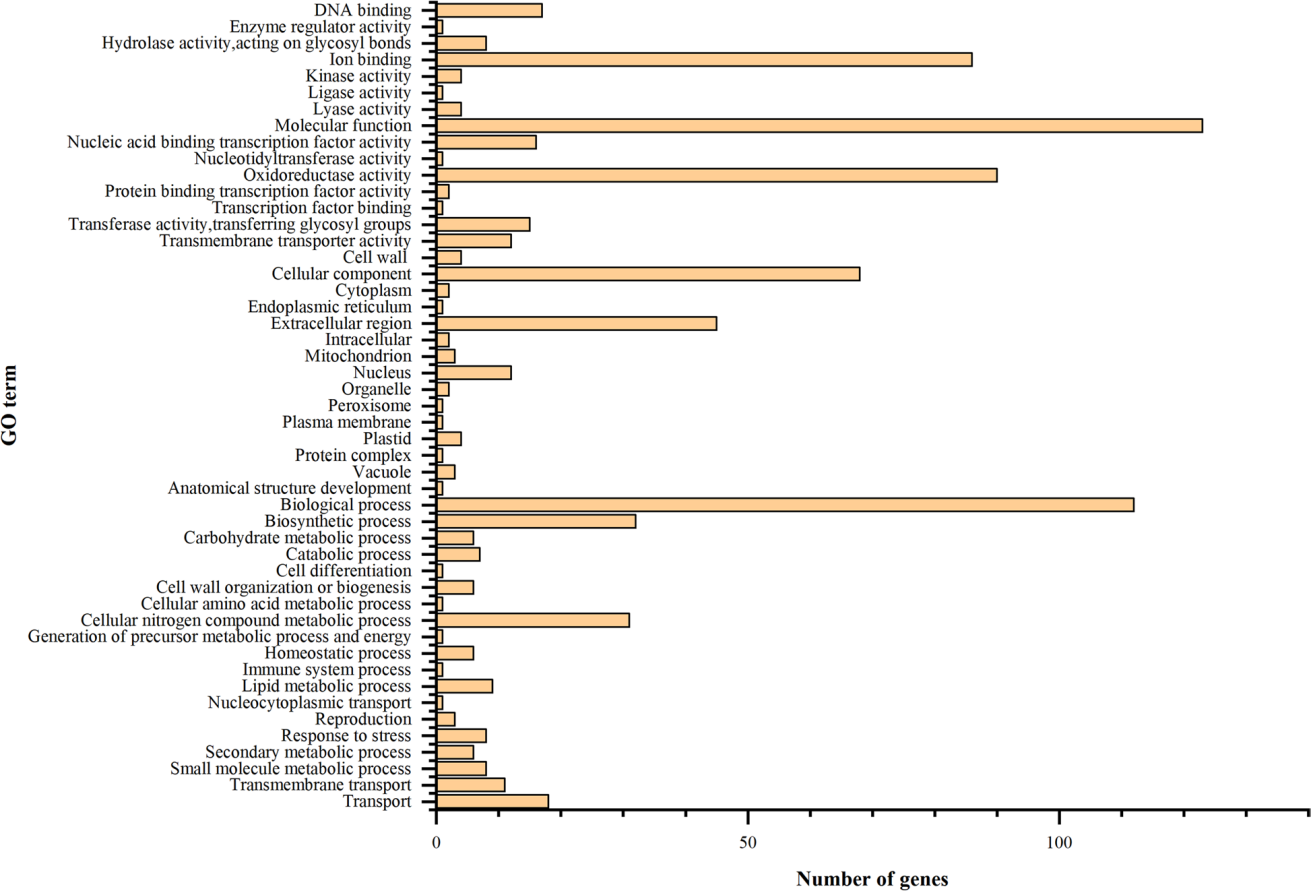
The most significantly-enriched GO terms of the differentially expressed genes from the four comparison groups: **B**: NNR vs CKR

Gene Ontology (GO) enrichment analysis indicated that the biological functions were significantly associated with differentially expressed transcripts. The DEGs in the NNL vs. CKL group separated into three main categories, including 60 GO groups based on biological process, 32 groups based on cellular component, and 39 groups based on molecular function (Fig. 6). However, The DEGs in the NNR vs. CKR group separated into three main categories, including 50 GO groups based on biological process, 29 groups based on cellular component, and 34 groups based on molecular function (Fig. 7). GO term enrichment analysis showed that 205 DEGs in tea leaf were enriched in seven GO terms based on *P* < 0.05. In these GO terms, 17 DEGs were enriched in photosystem I (GO:0,009,522), 9 DEGs were enriched in photosynthesis light harvesting (GO:0,009,765), 17 DEGs were enriched in chlorophyll binding (GO:0,016,168), 38 DEGS were enriched in chloroplast thylakoid membrane (GO:0,009,535, cellular component), 89 DEGs were enriched in transmembrane transport (GO:0,055,085), 17 DEGs were enriched in protein-chromophore linkage (GO:0,018,298, cellular component), 18 DEGs were enriched in transmembrane transporter activity (GO:0,022,857, cellular component), respectively (Table S5 (Supplementary Materials)). The expression levels of DEGs enriched in GO term Photosystem I (GO:0,009,522), Photosynthesis, light harvesting (GO:0,009,765), Chlorophyll binding (GO:0,016,168), and Chloroplast thylakoid membrane (GO:0,009,535)were down-regulated by N deficiency in tea leaves, except for K07573 exosome complex component CSL4 (TEA005899.1) and organic cation/carnitine transporter 4 (TEA004782.1).

GO enrichment analysis showed that 152 DEGs in tea roots were enriched in 11 GO items (*P* < 0.05). In these GO terms, 33 DEGs were enriched in extracellular region (GO:0,005,576), 27 were enriched in monooxygenase activity (GO:0,004,497), 15 DEGs were enriched in nutrient reservoir activity (GO:0,045,735), 24 DEGs were enriched in oxidoreductase activity (GO:0,016,705),38 DEGs were enriched in metabolic process (GO:0,008,152), 16 DEGs were enriched in manganese ion binding (GO:0,030,145), 31 DEGs were enriched in heme binding (GO:0,020,037), 30 DEGs were enriched in iron ion binding(GO:0,005,506), 12 DEGs were enriched in transferase activity (GO:0,016,758), 6 DEGs were enriched in oxidation–reduction process (GO:0,055,114),31 DEGs were enriched in transferase activity (GO:0,016,740), respectively (Table S6 (Supplementary Materials)). In order to identify the main biological processes and its related DEGs which were involved in tea transcriptomic response to N deficiency, hereafter we will focus our attention on the DEGs that were enriched GO terms with regard to biological processes, such as photosynthetic apparatus [photosystem I (GO:0,009,522),photosynthesis light harvesting (GO:0,009,765),chlorophyll binding (GO:0,016,168)], transmembrane transport (GO:0,055,085) in tea leaves, metabolic process(GO:0,008,152) and oxidation–reduction process (oxidoreductase activity, GO:0,016,705) in tea roots.

### Validation of RNA-Seq results by RT-qPCR

**Fig. 8:**
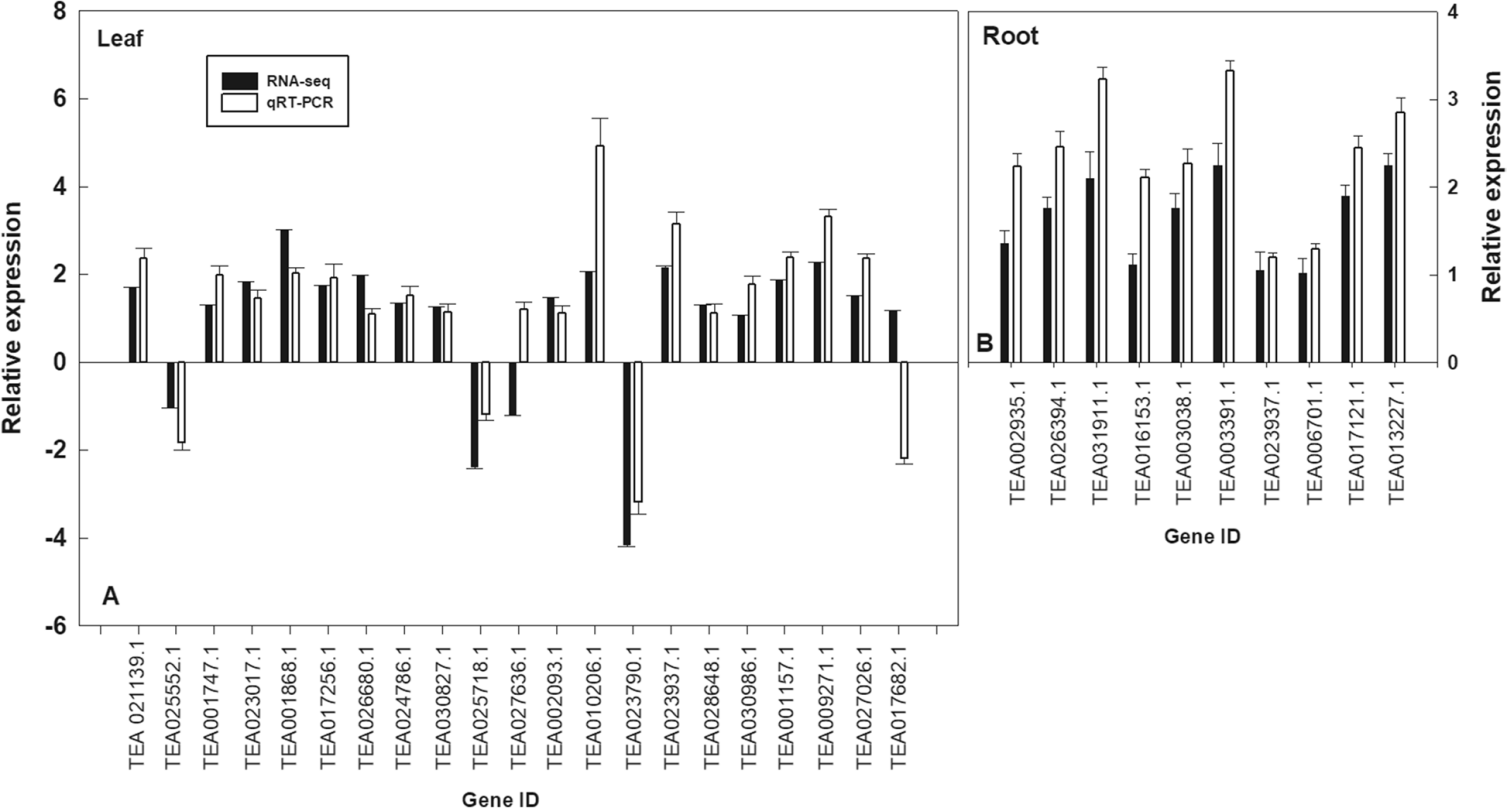
Relative expression levels of DEGs of N-deficient leaf compared to the control ones. The data were represented as mean of six biological replicates (*n* = 6). The GAPDH genes (CSA024857) were selected as the internal standards and the leaves from control seedlings were used as reference sample, which was set to 1

In order to verify the reliability of the RNA-seq results, 31 DEGs were randomly selected from RNA-seq data to perform RT-qPCR analysis. The RNA samples used for the RT-qPCR assay were processed in the same way as the RNA-seq samples. Except for TEA027636.1 and TEA017682.1, the expression patterns of the other 29 selected DEGs were highly consistent with the RNA-seq data, demonstrating that the RNA-seq data were robust and reliable method to isolate and identify N-deficiency responding DEGs in tea leaves (Fig. 8).

## Discussion

### Nitrogen-deficiency inhibited the growth, biosynthesis of Chl, Nmetabolism of tea plants

Nitrogen (N) is the key factor limiting the crop growth, acting as a constituent component of all proteins, nucleic acids and other organic compounds [1]. In the current study, N deficiency significantly decreased the DW and the N content of roots and leaves, the DW of stems and contents of Chl, but increased the ratio of root DW to shoot DW (Figs. 1 and 4). As one of most important macro-nutrients in plants, nitrogen deficiency led to growth-inhibition, yield loss, and deteriorating agricultural product quality in many corps, including sunflower, pea, rice, maize, tomato, tea plants and wheat [4, 6, 12, 27–31]. In the study, we observed that various sets of DEGs were responsive to N deficiency. The nitrogen-starved stress not only delayed sprouting of tea plants, but also significantly inhibited the growth of tea plants when they were compared with the control ones (Table 1; Fig. 1). In this case, the biomass of tea leaves would be apparently reduced by N deficiency. However, as a special commodity, the price of tea is also due in large part to the sensual qualities. The aroma, flavor and taste of tea products are mainly determined by the contents of amino acids, organic acids, soluble sugars and evaporating aromatic compounds, all of which are originated from the carbon skeletons generated by photosynthetic carbon assimilation, glycolytic pathway and the tricarboxylic acid cycle (TCA cycle), thus the decreased Chl content and subsequent leaf chlorosis lead to the reduction of photosynthesis, which eventually reduce the formation of flavor-related compounds in tea products [4, 32]. Recent study reported that high light, especially high ambient ultraviolet B (UV-B) increased bitter- and astringent-tasting flavonol glycosides, including kaempferol-7-O-glucoside, myricetin-3-O-glucoside, and quercetin-7-O-glucoside, but decreased catechins in tea leaves [33, 34]. In our study, we also found that N deficiency increased the content of total flavonoids in tea leaves (Fig. 2F). This result is in accordant with the finding that low N availability significantly increased the contents of quercetin, kaempferol and isorhamnetin in tomato leaf [31]. Therefore, we proposed that like high UV-B, N deficiency might also lower the quality of the tea products by increasing bitter- and astringent-tasting compounds.

To be incorporated into amino acids and peptides, nitrate is firstly reduced and converted into nitrite by nitrate reductase (NR) in the cell cytosol, and nitrite is then reduced and converted into ammonium by nitrite reductase (NiR) in plant plastids or leaf chloroplasts. Subsequently, glutamine synthetase (GS) incorporates ammonium into glutamine through the glutamine synthetase-glutamate synthase (GS-GOGAT) cycle, a crucial step for transforming inorganic nitrogen to organic nitrogen in plants [20]. Here, we found that the activity of NR and NiR were significantly down-regulated by N deficiency in tea plants (Figs. 2 and 3). Moreover, our results showed that N deficiency significantly reduced the activities of GOGAT (Figs. 2D and 3D) in tea roots and leaves, implying that N limitation might inhibit tea plant development via down-regulating GOGAT activities and related amino acid biosynthesis [35]. However, N deficiency increased the activity ofGS in tea leaves and roots (Figs. 2E and 3E). GS is an enzyme used by organisms to synthesize glutamine from other substances, such as glutamate. It is an important physiological indicator to measure the level of nitrogen assimilation in plants. The different change of GS compared with that of NR, NiR and GOGAT might be a strategic response to enhance the re-utilization of limit N resource under N deficiency condition.NR and NiR activities were regulated by many environmental factors including nitrate supply, amino acid level, and sugar level [21, 22]. Similarly, the down-regulation of NR and GOGAT activities by decreasing N supply was also observed in citrus [23]. Interestingly, in our RNA-seq data, we found that the expression pattern of DEGs encoding NR and GOGAT were in coincident with the enzyme activities of NiR and GOGAT in tea leaves under N deficient condition, whereas the expression pattern of DEGs encoding NR and GS were not in coincident with the enzyme activities of NR and GS in tea leaves under N deficient condition (Table S7). We proposed that the post-transcriptional regulation of NR and GSmight be the cause of this inconsistency.

### DEGs involved in photosynthetic apparatus in response to nitrogen-deficiency in tea plant

Nitrogen is an important constituent in RuBisCO protein, the Calvin–Benson cycle related enzymes, chlorophyll, and carotenoids in plant leaves [36]. N-deficiency-induced reduction of photosynthesis has been found in many crop including sunflower [13], rice [14], olive [15], and corn [16].The down-regulated expression levels of DEGs encoding photosystem I reaction center subunit N, light-harvesting complex I (LHCI), chlorophyll a/b binding protein, photosystem II protein D1 (psbA), ATP synthase CF1 beta subunit, oxygen-evolving complex (OEC) enhancer protein (TEA006799.1 and TEA027026.1), demonstrated that N deficiency might impair key functions from light-harvesting antenna pigments of photosystem II to the acceptor side of photosystem I. The light harvesting complex (LHC) proteins are members of a superfamily that can bind to chlorophyll and carotenoid to form pigment-protein complexes [37], which surround the photochemical reaction centers of PSI (LHCI) and PSII (LHCII) [38]. Four of the LHCII and two LHCI DEGs including TEA030284.1 (-2.76-fold),TEA028092.1 (-2.41-fold), TEA001864.1 (-2.21-fold),TEA001868.1 (-2.98-fold), TEA008208.1 (-2.36-fold),TEA016942.1 (-2.37-fold) were down-regulated by more than twofold in nitrogen deficiency leaf compared to the control ones. The results of the RT-qPCR analysis are consistent with the RNA-seq analysis (Fig. 8). The LHCs of land plants and green algae play an essential role in light capture and photo-protection [39, 40]. Thus, we proposed that the down-regulated LHC genes and lower CO_2_ assimilation in N deficient tea leaves might imply that N deficiency lower the light-capture ability in the photosynthetic membrane of tea plants when compared to N sufficient ones. Similarly, Shao et al. also found that N deficiency down-regulated most of the DEGs involved in photosynthesis and light harvesting process, such as *chlorophyll a/b binding proteins*, *photosystem I reaction center subunits*, *photosystem II reaction center subunits*, and so on, in rice leaves [14]. Previous studies reported that N deficiency inhibited photosynthetic capacity through decreased stromal and thylakoid proteins in transpiration, stomatal conductance, the chlorophyll and carotenoids contents [41, 42]. Thus, the current study provides a molecular basis for further understanding of the effects of N deficiency on the photosynthesis apparatus in tea leaves.

### DEGs involved in transport activities in response to nitrogen-deficiency in tea plant

In higher plants, the uptake of nitrate is mediated by two nitrate uptake systems: a low-affinity nitrate transport system and a high-affinity nitrate transport system [43]. Compared with other species, tea plant carries a much smaller set of nitrate transporter (NRT) genes [6]. The NRT1 and NRT2 families are involved in N translocation and utilization for plant growth [43]. Our results showed nitrite transporter 1 (NRT1) genes, including TEA021139.1 (-1.69-fold) and TEA029267.1 (-3.07-fold), were down-regulated by N deficiency in tea (Table S1). However, the gene expression level of a high affinity nitrate transporter 2.7 (TEA022890.1, *NRT2.7*) was up-regulated by N deficiency in tea leaves (Table S5). NRT2.7 is responsible for loading nitrate into plant vacuoles [20]. Such result might indicate that as a strategical response, the translocation of nitrate into leaf vacuoles was enhanced under N limited condition.

Besides NRT transporters, the ammonia transporters (AMT) were demonstrated to be responsive to N depletion at different developmental time points [44]. The expression level of *AMT* genes could be induced by nitrogen deficiency in *Arabidopsis* [45]. However, N deficiency decreased the gene expression level of several ammonium transporter genes, such as *AMT1* (TEA005896.1, 2.38-fold; TEA032584.1, 2.18-fold) and *AMT3* (TEĂ68.1, 1.01-fold) in the current study (Table S1). Similarly, the expression level of their homolog genes in tomato and green microalga were also repressed by N deprivation [46, 47]. These results implicated that AMT genes may play common and important roles in nitrogen assimilation in plants including tea. Here, the decreased gene expression level of DEGs related to nitrogen metabolism under N deficiency, such as *ferredoxin-nitrite reductase*, *NR* and *carbonic anhydrases*, indicated that the response of N metabolism to N deprivation was regulated at the transcriptional level. However, another study reported that the expression level of AMT genes was unaffected by N supply in cucumber [48]. Loqué et al. [49] proposed that AMT genes might have diverse expression patterns in different plant species, and did not serve as a universal biomarker in the nitrogen-deficient environment.

Recent studies reported that ATP-binding cassette (ABC) transporters transport secondary metabolites, supportive materials, and plant hormones that regulate plant growth, nutrition, development, and response to abiotic stress in plants. The category and protein abundance of ABC transporters were multiplied during plant evolution and assumed novel functions that allowed plants to adapt to environmental conditions [50]. Here, we found that the expression level of sixteen and six ABC transporter genes were up-regulated and down-regulated by N deficiency in tea leaves, respectively (Table S6), revealing cellular transport of secondary metabolites and plant hormone might be accelerated by N limitation in tea leaves. Accordingly, the up-regulated expression levels of ABC transporter gene were also reported in rice under heavy-metal stress [51], N-deficient condition [14], and *Citrus grandis* under aluminum stress [52]. Besides ABC transporters, the expression level of three organic cation/carnitine transporter 4 genes (TEA004782.1, TEA029454.1 and TEA029088.1), which involved in primary root growth and leaf development, and were also up-regulated by N deficiency in tea leaves (Table S5) [53]. The induced gene expression of *organic cation/carnitine transporter 4* suggested that mitochondrial and chloroplastic carnitine cycle could be enhanced in tea leaves under N deficiency [53].

Nucleobase-ascorbate transporters (NATs), belong to an evolutionarily widespread family of transport proteins and are known to transport xanthine and uric acid in plants. Their transcript levels were generally up-regulated significantly in response to the imposition of drought or salt stress [54]. Evidence showed that xanthine and uric acid have potential uses in salt stress alleviation, and the induced expression of *MdNAT* can enhance the concentration of xanthine and uric acid and improve the tolerance of salinity stress by increasing the activities of ROS scavenging enzymes, such as ascorbate peroxidase (APX) and glutathione reductase (GR) in apple [55]. Here, we found that the expression levels of two *NAT* genes, nucleobase-ascorbate transporter 6 (TEA031930.1) and nucleobase-ascorbate transporter 12 (TEA000826.1), were up-regulated by N deficiency in tea leaves (Table S5). This result indicated that similar as other environment stress, N deficiency could also up-regulate antioxidant system.

It is well known that the deficiency of one mineral nutrient leads to imbalance of nutrient proportion and influences the uptake of other nutrients [56]. In plant nutrition, there's quite obviously a lot of synergy among some nutrients. N deficiency decreased the content of P and up-regulated the expression levels of genes encoding inorganic P transporter 1–5, PHO1, and some genes involved in cellular response to phosphate starvation in rice [14, 57]. Conversely, P deficiency could also decrease the content of N in citrus leaves [58]. In the current study, we found that except for inorganic phosphate transporter 1–4 (TEA010724.1), the expression levels of four phosphate transporter genes, named probable anion transporter 3 (chloroplastic TEA015946.1, TEA004543.1), probable inorganic phosphate transporter 1–9 (TEA003831.1), and K14684 solute carrier family 25 (mitochondrial phosphate transporter) (TEA020321.1), were up-regulated by N deficiency in tea leaves (Table S5).

### DEGs involved in metabolic process in response to nitrogen-deficiency in tea roots

UDP-glycosyltransferases (UGTs) catalyze the glucose conjugation of monolignols, which is essential for normal cell wall lignification in plants [7]. In this study, we found that the UGT 73C1-like gene (TEA012722.1) was downregulated by N deficiency and the UGT 72C1 (TEA014953.1) gene was upregulated by N deficiency in tea roots (Table S6). Such results indicated that N deficiency might disturb the biosynthesis of lignin in tea root cells. In contrast, previous literatures showed that N deficiency strongly increased lignification of stem xylem and leaf tissue in tobacco and rice, respectively, by using microscopic observation, biochemical analysis and RNA-seq [14, 59]. Organic acids metabolism connects the carbon metabolism and amino acids metabolism. Here, we found two key genes [aconitate hydratase (TEA003717.1) and pyruvate dehydrogenase (TEA023576.1)] involved in TCA cycle were induced by N deficiency in tea roots (Table S6). This is in consistence with the result of our metabolomic analysis, which showed that N deficiency enhanced the TCA cycle in tea roots, revealing by increasing the contents of organic acids such as malic acid and citric acid [5]. The increased organic acids not only supplied sufficient carbon precursors for the amino acid biosynthesis, but also provided secretory solute to improve nutrient absorption efficiency in the rhizosphere of N or P-deficient tea roots [5, 60]. The secretion of scopoletin was considered as a responsive mechanism in plants under iron deficiency [61]. Here, we found that a gene encoding scopoletin glucosyltransferase (TEA008451.1) was downregulated by N deficiency, implying that the absorption of iron might be compromised in tea roots under N shortage (Table S6) [61, 62].

Plant hormones, such as ethylene, cytokinin and salicylic acid are closely associated with physiological and morphological responses to nutritional deficiency including P starvation, iron deficiency and K deficiency [63]. Under N deficiency, DEGs encoding a ethylene-responsive transcription factor RAP2-7-like (TEA001455.1), a cytokinin-O-glucosyltransferase 2 (TEA012394.1), and a salicylic acid-binding protein 2 (TEA008410.1), respectively, were downregulated by N limitation in tea roots, meaning that N deficiency had a global impact on root development and response to environmental stimuli via regulated plant signal transduction and regulation (Table S6). In addition, we found that genes encoding cyanohydrin beta-glucosyltransferase-like (TEA016859.1) and two tuliposide A-converting enzyme 2 (TEA029559.1 and TEA027973.1) were upregulated by N deficiency in tea roots (Table S6). Cyanogenic glycosides play pivotal roles in the organization of chemical defense system in plants and in plant–insect interactions [64]. Tuliposide A, a plant metabolite, has antibacterial and antifungal properties [65]. The upregulated gene expression of cyanohydrin beta-glucosyltransferase-like and tuliposide A-converting enzyme 2 might increase the tolerance of adverse conditions under N limitation.

### DEGs involved in oxidation–reduction process in response to nitrogen-deficiency in tea roots

In plants, lipoxygenase (LOX) catalyzes the conversion of polyunsaturated fatty acids into conjugated hydroperoxides. LOX is considered to be involved in jasmonic acid biosynthesis and be in response to biotic and abiotic stresses [66]. A DEG (TEA011776.1) encoding lipoxygenase isoform 2 was downregulated by N deficiency in tea roots (Table S6), demonstrating that the biosynthesis of jasmonic acid might be compromised in N-deficient tea roots. In contrast, previous literatures showed that both B-deficiency and High C: Low N treatment enhanced the protein abundance or gene expression level of LOX2 in citrus roots and rice roots, respectively [66, 67].

Carotenoid cleavage dioxygenases (CCDs) are non-haem iron oxygenases that cleave carotenes and xanthophylls to apocarotenoids. These substances are widely distributed in nature and have important metabolic and hormonal functions in plants [68]. It is well known that certain apocarotenoids act as hormones in the regulation of plant growth. The best characterized examples are abscisic acid (ABA) and strigolactones, which play an important role in the regulation of stress tolerance, including drought and nutrient shortage [29, 68, 69]. In present study, the increased expression levels of three DEGs (TEA017638.1, TEA024310.1 and TEA028072.1) encoding CCDs by N deficiency were observed in tea roots (Table S6). However, two DEGs encoding 9-cis-epoxycarotenoid dioxygenase (NCED), which catalyzed the final step of abscisic-acid biosynthesis, were differentially regulated by N deficiency in tea roots, with one upregulated and another one downregulated (Table S6) [14, 70]. Such results indicated that N deficiency might heterogeneously regulate the metabolism of plant hormone, at least ABA, in tea roots.

## Conclusions

N-deficiency significantly decreased the content of N and biomass of roots and leaves, and delayed the sprouting time of tea buds. N-deficiency-induced decreased content of Chl and decreased activities of N metabolism related enzymes, such as NR, NiR and GOGAT in tea leaves and roots, might play a part in the retardation of tea growth under N deficiency. By using RNA-seq technique and subsequent bioinformatics analysis, a total of 5738 and 1769DEGs were isolated in tea leaves and roots, respectively, in response to N deficiency. GO term enrichment analysis showed that 205 DEGs in tea leaves were enriched in seven GO terms and 152 DEGs in tea roots were enriched in 11 GO items at *P* < 0.05. In tea leaves, most of GO-enriched DEGs were involved in chlorophyll *a*/*b* binding activities, photosynthetic performance and transport activities in tea leaves. But most of DEGs in tea roots were involved in the metabolism of carbohydrate and plant hormone with regard to the GO terms of biological processes. It is worth mentioning that N deficiency significantly increased the expression level of phosphate transporter genes, which indicated that N deficiency might impair phosphorus metabolism in tea leaves. In conclusion, this study increased our knowledge about physiological and molecular mechanisms of tea plant in response to N limited condition. Furthermore, some DEGs in response to N deficiency, such as *NATs*, *probable anion transporter 3*, *organic cation/carnitine transporter 4*, and *high affinity nitrate transporter 2.7*, could be used to improve the tolerance of N deficiency in tea plants by genetic modification in future study.

## Materials and methods

### Plant culture and Ntreatement

The cultivation of tea plants and different N treatments were carried out according to the method described by Lin et al. [7]. Tea seedlings were raised from cuttings of cultivar “Chunlv2” [*Camellia sinensis* (L.) O. Kuntze], which was bred from tea cultivar “Fuyun6” by Tea Research Institute, Fujian Academy of Agricultural Sciences (Fu’an, China) [5].We transplanted the uniform 10-month-old tea plants to 6 L pottery pots (two plants per pot) containing clean river sands and treated with nutrient solutions with 0 mM NH_4_NO_3_ (N-deficiency) or 3 mM NH_4_NO_3_ (control)until dipping (about 500 mL) every two days. The tea plants were cultivated under natural light at Tea Research Institute, Fujian Academy of Agricultural Sciences. Four months later, new shoots with one bud and two leaves were punched and root apices (about 5 mm) were collected at noon on a sunny day. These samples were frozen by liquid nitrogen and stored at -80˚C until the determination of RNA-seq and physiological components.

### Determination of dry weight, nitrogen content, total flavonoids,chlorophyll, polyphenols and L-theanine contents

Plant samples were dried at 108˚C for 48 h, and then weighed. The samples were digested by roots, stems and shoots to measure N content using H_2_SO_4_-H_2_O_2_ and a continuous flow auto-analyzer AAIII (SEAL Analytical, Germany).

The flavonoid was extracted and measured according to the method described by Krizek et al. [71]. Absorbances were examined at three wavelengths: 270, 300 and 330 nm. Leaf chlorophyll (Chl) was measured according to the method described by Lichtenthaler [72] and Li et al. [73]. In brief, leaf Chls were extracted with 80% acetone (v/v) solution and measured at the wavelength of 663 nm, 645 nm and 470 nm, respectively, using Libra S22 ultraviolet–visible spectrophotometer (Biochrom Ltd, Cambridge, UK). Polyphenols were extracted and measured using Folin-Ciocalteu reagent with gallic acid as standard. L-theanine was extracted by deionized water and measured using high performance liquid chromatography (HPLC) (Agilent 1260 Infinity, Agilent Technologies, Santa Clara, USA).

### Measurement of spring shoot growth period

The spring shoot growth period were determined by the method described byChen et al. [74]. In brief, the growing period of one bud with the first leaf, one bud with the second leaf and one bud with the third leaf were observed and recorded. Before the tea plants sprout, ten buds (from different pots) of each treatment were randomly selected for regular observation every day. As long as 30% of observed buds reached the phenology standard, the growth time of this stage was recorded.

### Determination of N metabolism related enzyme assays

The leaves or roots were ground with a pre-cooled mortar and pestle in 2 mL ice-cold extraction buffer containing 50 mmol L^−1^ K_2_PO_4_-KOH (pH 7.5), 2 mmol L^−1^ EDTA, 1.5% (w/v) soluble casein, 2 mmol L^−1^ dithiothreitol(DTT) and 2% (w/v) insoluble polyvinylpolypyrrolidone (PVPP).The homogenate was centrifuged at 15000* g* at 4 °C for 20 min. Supernatantwas used to measure enzyme activity of NR, NiR and GOGAT (Singh and Srivastava 1986). The activity of NR was measured according to the method described by Rubio-Wilhelmia et al. [75] and Chen et al. [23]. The activity of NiR was measured according to themethod described byLillo [76]. The activity of GOGAT was assayed spectrophotometrically at 30 °C by monitoring theoxidation of NADH at 340 nm [77].The activity ofGS was determined using the hydroxamate synthetase assay described by Rubio-Wilhelmia et al. [75]. Briefly, leaf samples were homogenized with 50 mmol L^−1^ of maleic acid-KOH buffer (pH = 6.8) containing 100 mmol L^−1^ sucrose, 2% (v/v) β-mercaptoethanol and 20% (v/v) ethyleneglycol. The homogenate was centrifuged at 30,000 *g*at 4 °C for 20 min. The formation of glutamylhydroxamate was monitored at 540 nm after being chelated with acidified ferric chloride [78].

### RNA extraction and Illumina sequencing

The total RNA was extracted from frozen leaf and root samples using Plant Total RNA Extraction Kit (Tin Gen Biochemical Technology Co., Ltd. China) by following manufacturer’s instructions. Ten mg (500 ng mL^−1^) of high quality (the ratio of A260 nm/A280 nm was between 1.8 and 2.0) total RNA per sample was delivered to the Bena Biotechnology Corporation (Wuhan, China) for sequencing and generation of datasets. Magnetic Oligo (dT) beads (Illumina) were used to isolate poly (A) mRNA, and then fragmented into short fragments (200 nt) and synthesize first-strand cDNA using random hexamer-primers (N6) (Illumina). Second-strand cDNA was synthesized using 5 × buffer, 10 mmol L^−1^ each dNTPs, 2 U RNase H, and 40 U DNA polymerase I. Illumina GA Pipeline (Version 1.6) was used to perform the original image process to sequences, basecalling and quality value calculation, in which 125 bp paired-end reads were obtained. RNA-Seq reads were aligned to the tea genomes data [1].

### Sequence process, differentially expressed genes (DEGs) identification, and geneannotation

High-quality clean reads were obtained by filtering out adaptor sequences, reads containing poly-Ns and with low-quality. The sequence duplication level of the clean data and the Q20, Q30 and GC-contents were calculated.

Expression levels for each gene were calculated by quantifying the Illumina reads according to the FPKM (fragments per kilobase of transcript per million mapped reads) algorithm [79]. Replicates were examined independently for statistical analysis. Genes with at least two-fold expression change were considered as DEGs after being tested for false discovery rate (FDA) correlations at adjusted *p-*values ≤ 0.05. Functional annotation of DEGs was carried out by the method described by Young et al. [80] and Kanehisa*et al*. [81].

### Real-time quantitative RT-PCR (RT-qPCR) analysis of selected DEGs in response to N deficiency

To validate the results of the RNA-Seq data, a total of twenty-nine unigenes were randomly selected for RT-qPCR analysis. The RT-qPCR primer pairs were designed using Primer Premier 5.0 (Premier Biosoft, Palo Alto, CA, USA), and *GAPDH* (CSA024857.1) was set as the internal reference gene (Table S8). Total RNA was purified with an RNA purification kit (Tiangen, China) and first-strand cDNA synthesis was used the PrimeScript II 1^st^ Strand cDNA Synthesis Kit from TaKaRa (Dalian, China) according to the manufacturer’s instructions. Reactions were carried out using a SYBR Premix Ex Taq II (Tli RnaseH Plus) Kit (TaKaRa, Japan) in a Lightcycler 480 Real-Time PCR Detection System (Roche, Germany). Real-time qPCR was performed in a mixture containing 3 μL of template cDNA, 1 μL of forward primer (5 pmol), 1 μL of reverse primer (5 pmol), and 5 μL of SYBR Green mix (Qiagen, Hilden, Germany). The PCR processes included 5 min of pre-heat at 95 °C, 45 cycles of 10 s at 95 °C and 20 s at 60 °C, followed by steps of dissociation curve generation (15 s at 95 °C, 60 s at 60 °C, and 15 s at 95 °C). Each experiment was performed with three replicates, and the relative expression level of each gene was calculated using the 2 ^–ΔΔCt^ method. For the normalization of different samples, the *GAPDH* gene (accession number: CSA024857.1) were selected as the internal standards and the leaves from CK were used as reference sample, whose gene expression level was set to 1.

### Statistical analysis

There were 40 pots (80 plants) of each treatment in a completely randomized design. Each experiment was conducted by three to six replicates. Data were presented as mean ± standard error (*n* = 3–6). Significant difference between or among means was tested by student’s *t*-test or the least significant difference (LSD) test at *p* < 0.05 using SPSS software (Version 17.0, SPSS Inc., Chicago, IL, USA).The histograms were generated using Sigmaplot software (Version 10.0) and Venn diagram was drawn using Venny 2.1.0 (https://bioinfogp.cnb.csic.es/tools/venny/index.html).

## Supplementary Information

**Additional file 1: Table S1.** Down-regulated DEGs in response to N deficiency in tea leaf and root.

**Additional file 2: Table S2.** Up-regulated DEGs in response to N deficiency in tea leaf and root.

**Additional file 3: Table S3.** Down-regulated DEGs in response to N deficiency in tea roots.

**Additional file 4: Table S4.** Up-regulated DEGs in response to N deficiency in tea roots.

**Additional file 5: Table S5.** Enriched GO terms of DEGs in tea leaves and root under N deficiency.

**Additional file 6: Table S6.** Enriched GO terms of DEGs in tea roots under N deficiency.

**Additional file 7: Table S7.** DEGs involved in N metabolism in tea leaves under N deficiency.

**Additional file 8: Table S8.** Gene special primer pair using for RT-qPCR analysis.

## Data Availability

The RNA-Seq data was deposited in NCBI database (https://www.ncbi.nlm.nih.gov/sra/) with SRA accession number PRJNA747801.

## Abbreviations

N: Nitrogen
NR: Nitrate reductase
NiR: Nitrite reductase
GS: Glutamine synthetase
GOGAT: Glutamate synthase
Chl: Chlorophyll
